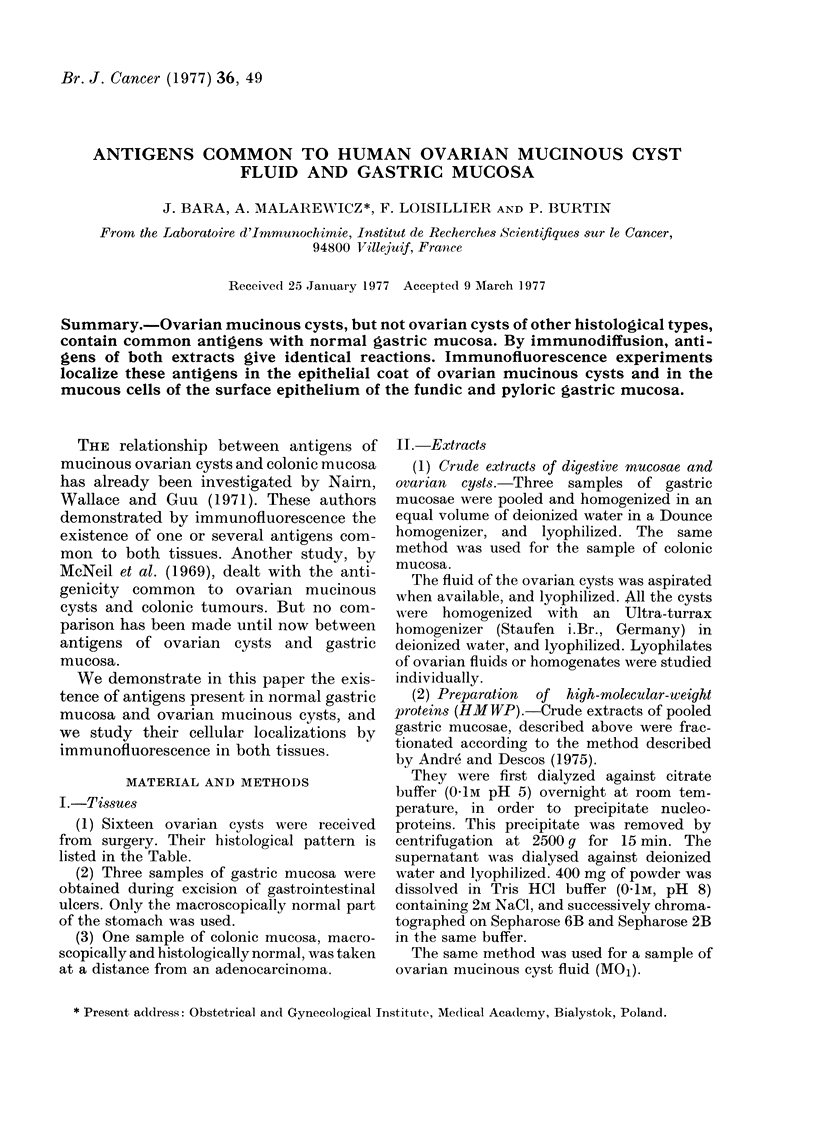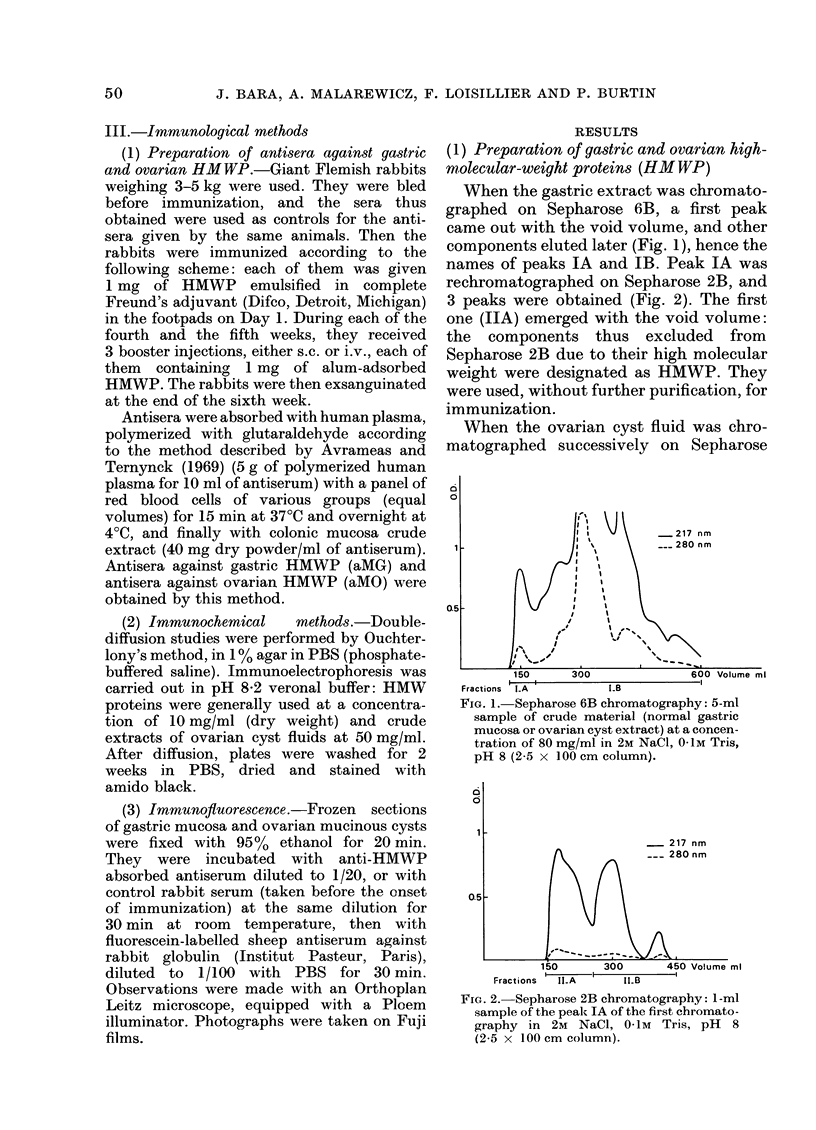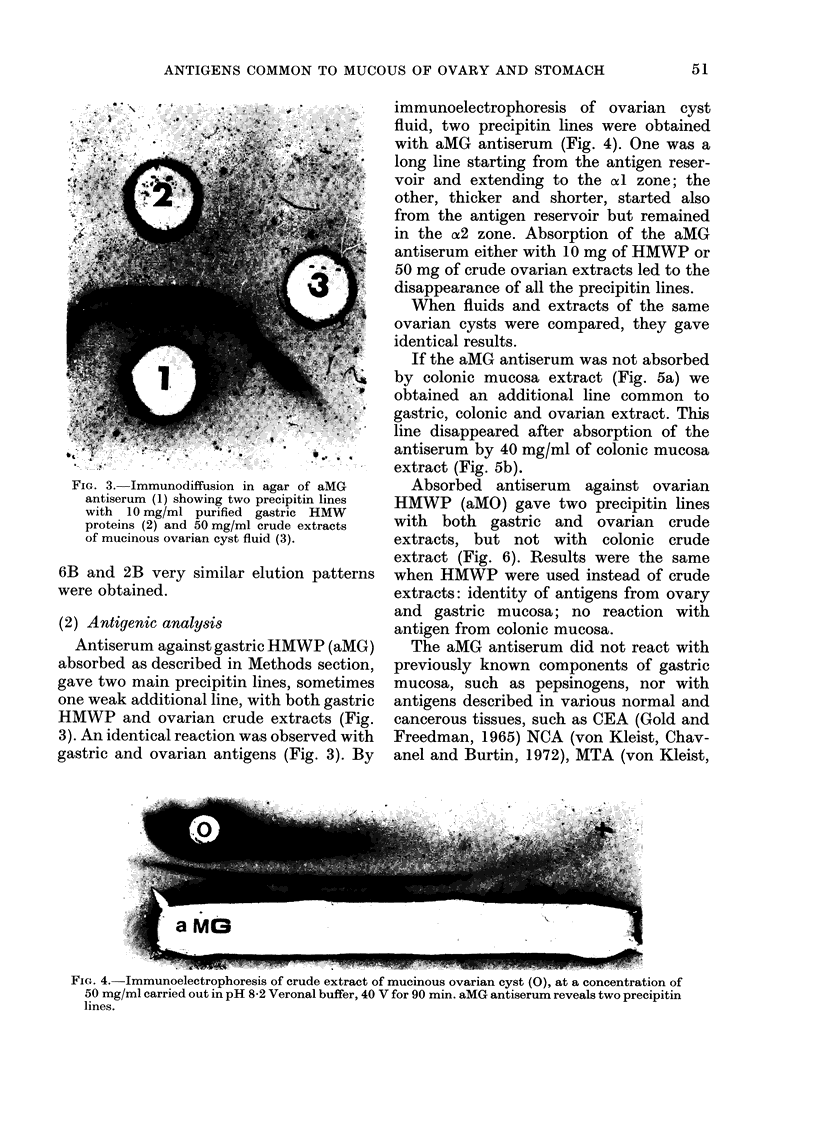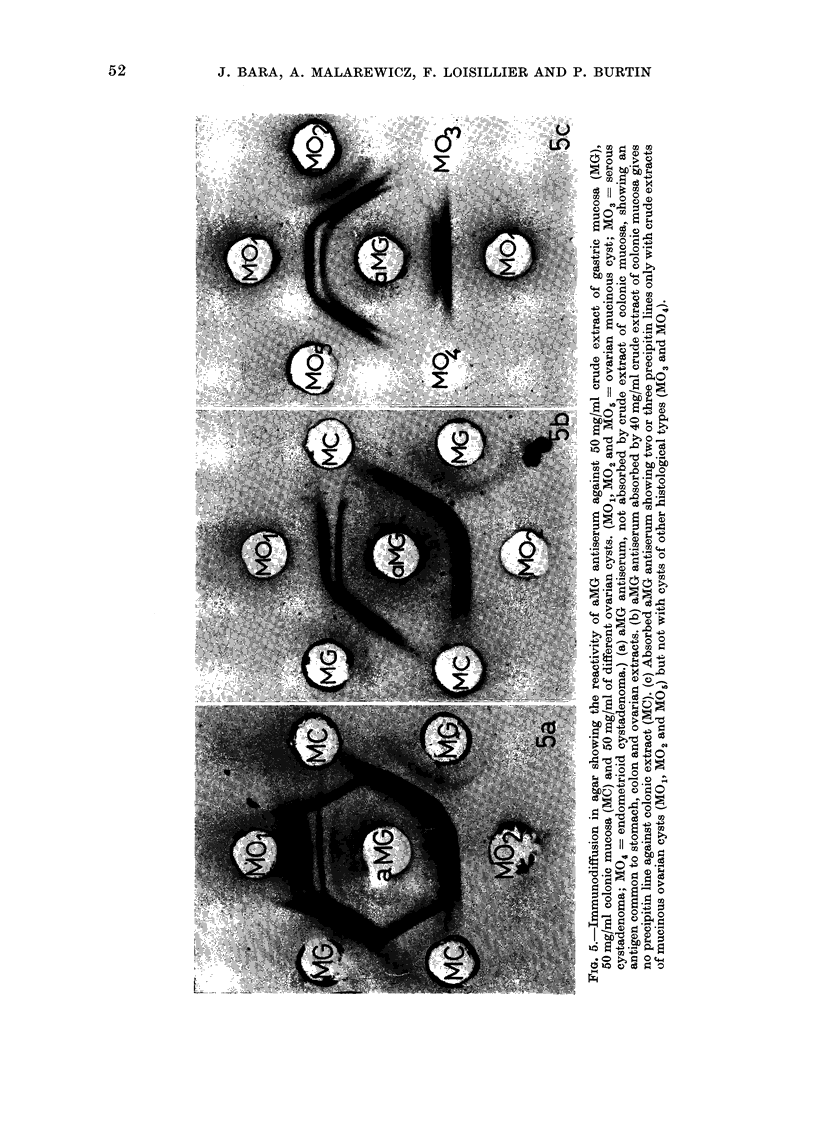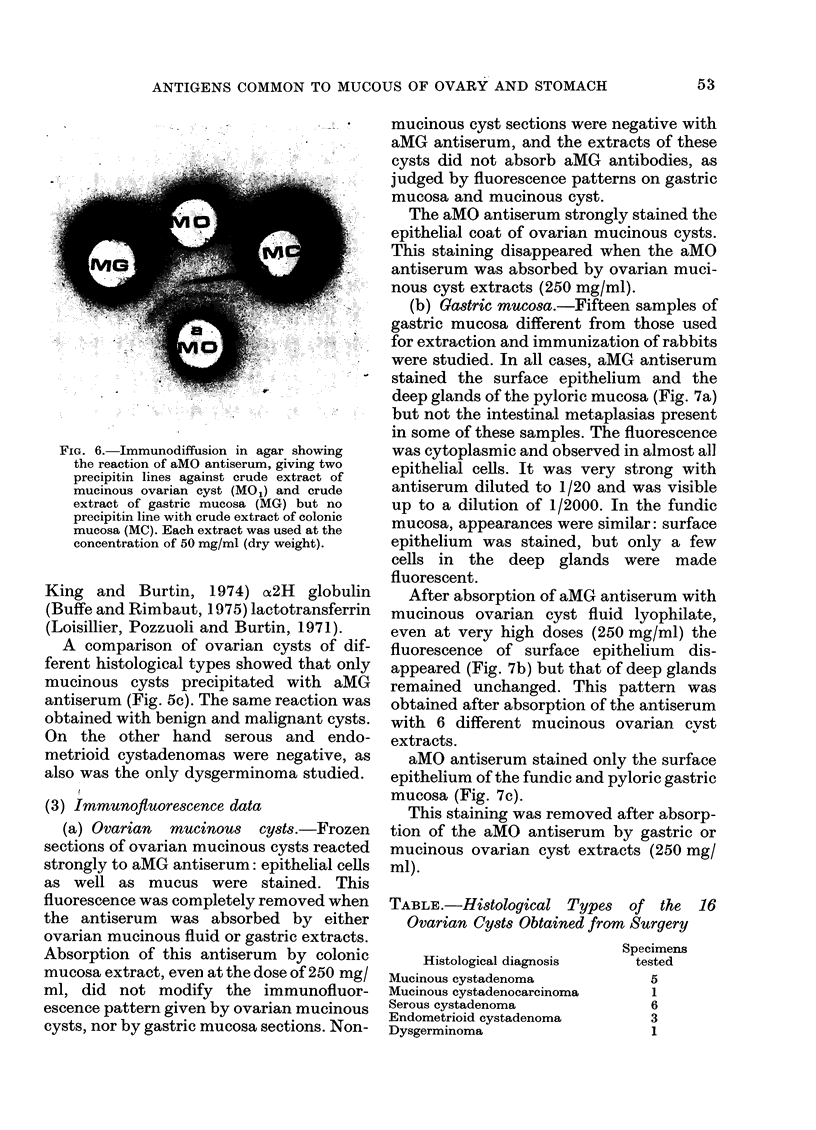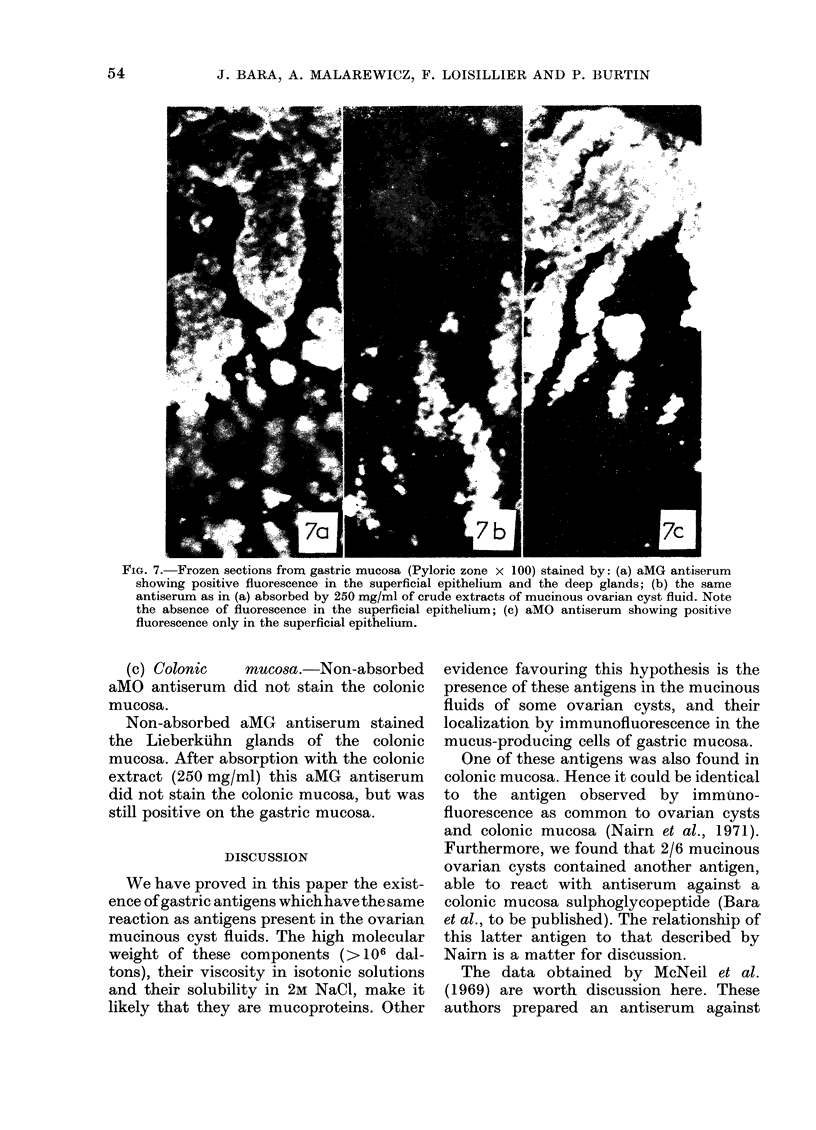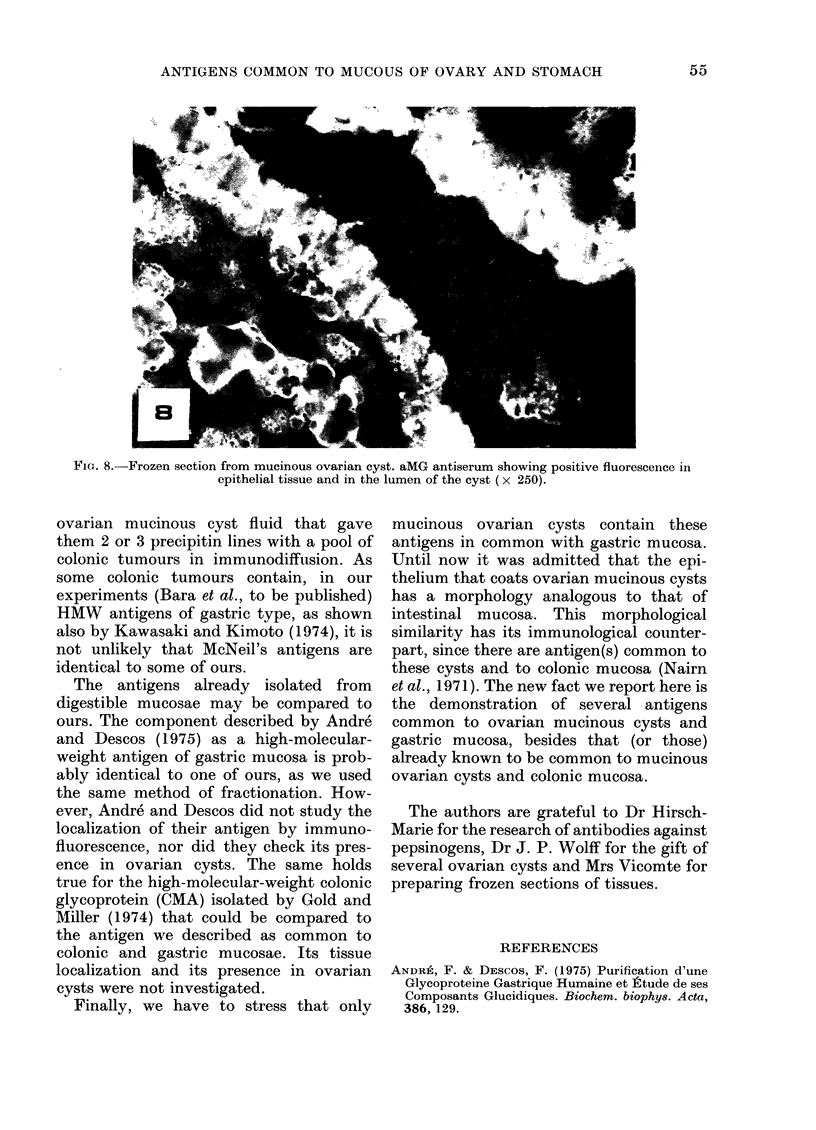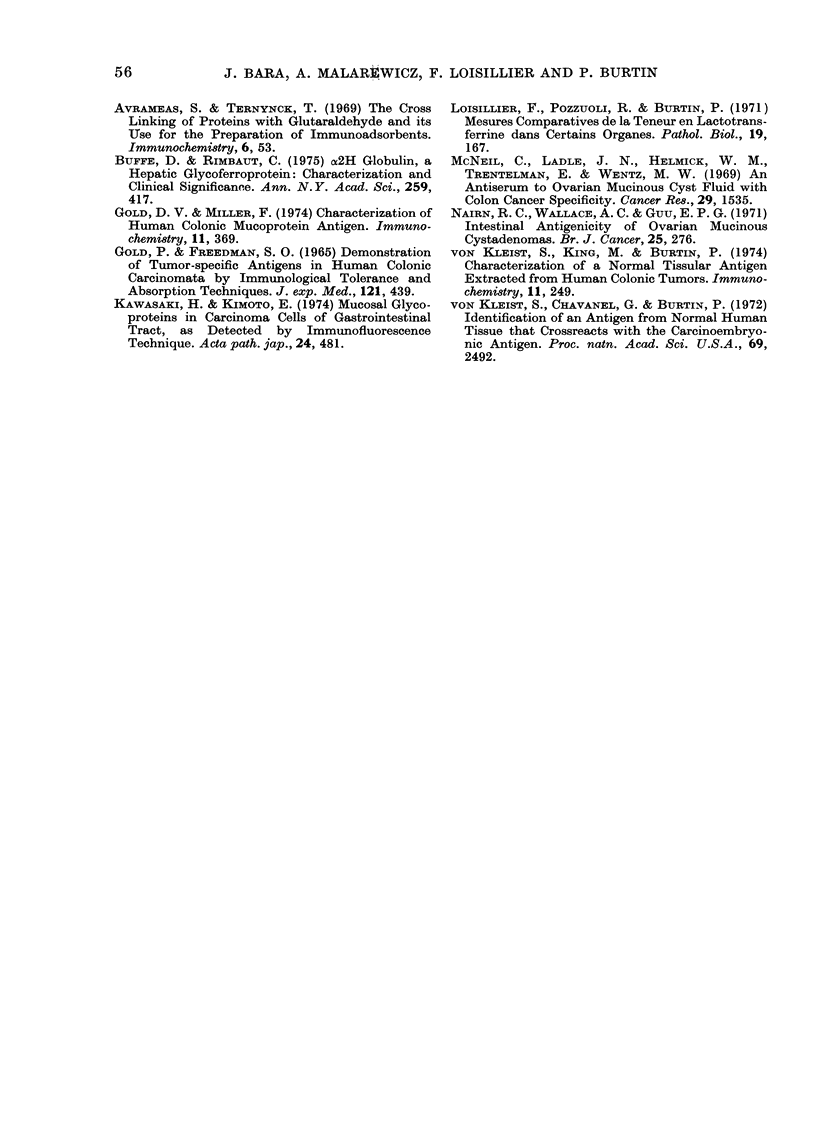# Antigens common to human ovarian mucinous cyst fluid and gastric mucosa.

**DOI:** 10.1038/bjc.1977.153

**Published:** 1977-07

**Authors:** J. Bara, A. Malarewicz, F. Loisillier, P. Burtin

## Abstract

**Images:**


*Br. J. Cancer* (1977) **36**, 49

### ANTIGENS COMMON TO HUMAN OVARIAN MUCINOUS CYST FLUID AND GASTRIC MUCOSA

J. BARA, A. MALAREWICZ*, F. LOISILLIER AND P. BURTIN

*From the Laboratoire d'Immunochimie, Institut de Recherches Scientifiques sur le Cancer, 94800 Villejuif, France*

Received 25 January 1977 Accepted 9 March 1977

**Summary.—Ovarian mucinous cysts, but not ovarian cysts of other histological types, contain common antigens with normal gastric mucosa. By immunodiffusion, antigens of both extracts give identical reactions. Immunofluorescence experiments localize these antigens in the epithelial coat of ovarian mucinous cysts and in the mucous cells of the surface epithelium of the fundic and pyloric gastric mucosa.**

THE relationship between antigens of mucinous ovarian cysts and colonic mucosa has already been investigated by Nairn, Wallace and Guu (1971). These authors demonstrated by immunofluorescence the existence of one or several antigens common to both tissues. Another study, by McNeil *et al.* (1969), dealt with the antigenicity common to ovarian mucinous cysts and colonic tumours. But no comparison has been made until now between antigens of ovarian cysts and gastric mucosa.

We demonstrate in this paper the existence of antigens present in normal gastric mucosa and ovarian mucinous cysts, and we study their cellular localizations by immunofluorescence in both tissues.

### MATERIAL AND METHODS

### I.—*Tissues*

(1) Sixteen ovarian cysts were received from surgery. Their histological pattern is listed in the Table.

(2) Three samples of gastric mucosa were obtained during excision of gastrointestinal ulcers. Only the macroscopically normal part of the stomach was used.

(3) One sample of colonic mucosa, macroscopically and histologically normal, was taken at a distance from an adenocarcinoma.

### II.—*Extracts*

(1) *Crude extracts of digestive mucosae and ovarian cysts.*—Three samples of gastric mucosae were pooled and homogenized in an equal volume of deionized water in a Dounce homogenizer, and lyophilized. The same method was used for the sample of colonic mucosa.

The fluid of the ovarian cysts was aspirated when available, and lyophilized. All the cysts were homogenized with an Ultra-turrax homogenizer (Staufen i.Br., Germany) in deionized water, and lyophilized. Lyophilates of ovarian fluids or homogenates were studied individually.

(2) *Preparation of high-molecular-weight proteins (HMWP).*—Crude extracts of pooled gastric mucosae, described above were fractionated according to the method described by André and Descos (1975).

They were first dialyzed against citrate buffer (0·1M pH 5) overnight at room temperature, in order to precipitate nucleoproteins. This precipitate was removed by centrifugation at 2500 *g* for 15 min. The supernatant was dialysed against deionized water and lyophilized. 400 mg of powder was dissolved in Tris HCl buffer (0·1M, pH 8) containing 2M NaCl, and successively chromatographed on Sepharose 6B and Sepharose 2B in the same buffer.

The same method was used for a sample of ovarian mucinous cyst fluid ($MO_1$).

* Present address: Obstetrical and Gynecological Institute, Medical Academy, Bialystok, Poland.

III.—*Immunological methods*

(1) *Preparation of antisera against gastric and ovarian HMWP.*—Giant Flemish rabbits weighing 3–5 kg were used. They were bled before immunization, and the sera thus obtained were used as controls for the antisera given by the same animals. Then the rabbits were immunized according to the following scheme: each of them was given 1 mg of HMWP emulsified in complete Freund's adjuvant (Difco, Detroit, Michigan) in the footpads on Day 1. During each of the fourth and the fifth weeks, they received 3 booster injections, either s.c. or i.v., each of them containing 1 mg of alum-adsorbed HMWP. The rabbits were then exsanguinated at the end of the sixth week.

Antisera were absorbed with human plasma, polymerized with glutaraldehyde according to the method described by Avrameas and Ternynck (1969) (5 g of polymerized human plasma for 10 ml of antiserum) with a panel of red blood cells of various groups (equal volumes) for 15 min at 37°C and overnight at 4°C, and finally with colonic mucosa crude extract (40 mg dry powder/ml of antiserum). Antisera against gastric HMWP (aMG) and antisera against ovarian HMWP (aMO) were obtained by this method.

(2) *Immunochemical methods.*—Double-diffusion studies were performed by Ouchterlony's method, in 1% agar in PBS (phosphate-buffered saline). Immunoelectrophoresis was carried out in pH 8·2 veronal buffer: HMW proteins were generally used at a concentration of 10 mg/ml (dry weight) and crude extracts of ovarian cyst fluids at 50 mg/ml. After diffusion, plates were washed for 2 weeks in PBS, dried and stained with amido black.

(3) *Immunofluorescence.*—Frozen sections of gastric mucosa and ovarian mucinous cysts were fixed with 95% ethanol for 20 min. They were incubated with anti-HMWP absorbed antiserum diluted to 1/20, or with control rabbit serum (taken before the onset of immunization) at the same dilution for 30 min at room temperature, then with fluorescein-labelled sheep antiserum against rabbit globulin (Institut Pasteur, Paris), diluted to 1/100 with PBS for 30 min. Observations were made with an Orthoplan Leitz microscope, equipped with a Ploem illuminator. Photographs were taken on Fuji films.

RESULTS

(1) *Preparation of gastric and ovarian high-molecular-weight proteins (HMWP)*

When the gastric extract was chromatographed on Sepharose 6B, a first peak came out with the void volume, and other components eluted later (Fig. 1), hence the names of peaks IA and IB. Peak IA was rechromatographed on Sepharose 2B, and 3 peaks were obtained (Fig. 2). The first one (IIA) emerged with the void volume: the components thus excluded from Sepharose 2B due to their high molecular weight were designated as HMWP. They were used, without further purification, for immunization.

When the ovarian cyst fluid was chromatographed successively on Sepharose

FIG. 1.—Sepharose 6B chromatography: 5-ml sample of crude material (normal gastric mucosa or ovarian cyst extract) at a concentration of 80 mg/ml in 2M NaCl, 0·1M Tris, pH 8 (2·5 × 100 cm column).

FIG. 2.—Sepharose 2B chromatography: 1-ml sample of the peak IA of the first chromatography in 2M NaCl, 0·1M Tris, pH 8 (2·5 × 100 cm column).

FIG. 3.—Immunodiffusion in agar of aMG antiserum (1) showing two precipitin lines with 10 mg/ml purified gastric HMW proteins (2) and 50 mg/ml crude extracts of mucinous ovarian cyst fluid (3).

6B and 2B very similar elution patterns were obtained.

(2) *Antigenic analysis*

Antiserum against gastric HMWP (aMG) absorbed as described in Methods section, gave two main precipitin lines, sometimes one weak additional line, with both gastric HMWP and ovarian crude extracts (Fig. 3). An identical reaction was observed with gastric and ovarian antigens (Fig. 3). By immunoelectrophoresis of ovarian cyst fluid, two precipitin lines were obtained with aMG antiserum (Fig. 4). One was a long line starting from the antigen reservoir and extending to the $\alpha 1$ zone; the other, thicker and shorter, started also from the antigen reservoir but remained in the $\alpha 2$ zone. Absorption of the aMG antiserum either with 10 mg of HMWP or 50 mg of crude ovarian extracts led to the disappearance of all the precipitin lines.

When fluids and extracts of the same ovarian cysts were compared, they gave identical results.

If the aMG antiserum was not absorbed by colonic mucosa extract (Fig. 5a) we obtained an additional line common to gastric, colonic and ovarian extract. This line disappeared after absorption of the antiserum by 40 mg/ml of colonic mucosa extract (Fig. 5b).

Absorbed antiserum against ovarian HMWP (aMO) gave two precipitin lines with both gastric and ovarian crude extracts, but not with colonic crude extract (Fig. 6). Results were the same when HMWP were used instead of crude extracts: identity of antigens from ovary and gastric mucosa; no reaction with antigen from colonic mucosa.

The aMG antiserum did not react with previously known components of gastric mucosa, such as pepsinogens, nor with antigens described in various normal and cancerous tissues, such as CEA (Gold and Freedman, 1965) NCA (von Kleist, Chavanel and Burtin, 1972), MTA (von Kleist,

FIG. 4.—Immunoelectrophoresis of crude extract of mucinous ovarian cyst (O), at a concentration of 50 mg/ml carried out in pH 8·2 Veronal buffer, 40 V for 90 min. aMG antiserum reveals two precipitin lines.

FIG. 6.—Immunodiffusion in agar showing the reaction of aMO antiserum, giving two precipitin lines against crude extract of mucinous ovarian cyst ($MO_1$) and crude extract of gastric mucosa (MG) but no precipitin line with crude extract of colonic mucosa (MC). Each extract was used at the concentration of 50 mg/ml (dry weight).

King and Burtin, 1974) α2H globulin (Buffe and Rimbaut, 1975) lactotransferrin (Loisillier, Pozzuoli and Burtin, 1971).

A comparison of ovarian cysts of different histological types showed that only mucinous cysts precipitated with aMG antiserum (Fig. 5c). The same reaction was obtained with benign and malignant cysts. On the other hand serous and endometrioid cystadenomas were negative, as also was the only dysgerminoma studied.

(3) *Immunofluorescence data*

(a) *Ovarian mucinous cysts.*—Frozen sections of ovarian mucinous cysts reacted strongly to aMG antiserum: epithelial cells as well as mucus were stained. This fluorescence was completely removed when the antiserum was absorbed by either ovarian mucinous fluid or gastric extracts. Absorption of this antiserum by colonic mucosa extract, even at the dose of 250 mg/ml, did not modify the immunofluorescence pattern given by ovarian mucinous cysts, nor by gastric mucosa sections. Non-mucinous cyst sections were negative with aMG antiserum, and the extracts of these cysts did not absorb aMG antibodies, as judged by fluorescence patterns on gastric mucosa and mucinous cyst.

The aMO antiserum strongly stained the epithelial coat of ovarian mucinous cysts. This staining disappeared when the aMO antiserum was absorbed by ovarian mucinous cyst extracts (250 mg/ml).

(b) *Gastric mucosa.*—Fifteen samples of gastric mucosa different from those used for extraction and immunization of rabbits were studied. In all cases, aMG antiserum stained the surface epithelium and the deep glands of the pyloric mucosa (Fig. 7a) but not the intestinal metaplasias present in some of these samples. The fluorescence was cytoplasmic and observed in almost all epithelial cells. It was very strong with antiserum diluted to 1/20 and was visible up to a dilution of 1/2000. In the fundic mucosa, appearances were similar: surface epithelium was stained, but only a few cells in the deep glands were made fluorescent.

After absorption of aMG antiserum with mucinous ovarian cyst fluid lyophilate, even at very high doses (250 mg/ml) the fluorescence of surface epithelium disappeared (Fig. 7b) but that of deep glands remained unchanged. This pattern was obtained after absorption of the antiserum with 6 different mucinous ovarian cyst extracts.

aMO antiserum stained only the surface epithelium of the fundic and pyloric gastric mucosa (Fig. 7c).

This staining was removed after absorption of the aMO antiserum by gastric or mucinous ovarian cyst extracts (250 mg/ml).

TABLE.—*Histological Types of the 16 Ovarian Cysts Obtained from Surgery*

| Histological diagnosis | Specimens tested |
|---|---|
| Mucinous cystadenoma | 5 |
| Mucinous cystadenocarcinoma | 1 |
| Serous cystadenoma | 6 |
| Endometrioid cystadenoma | 3 |
| Dysgerminoma | 1 |

FIG. 7.—Frozen sections from gastric mucosa (Pyloric zone × 100) stained by: (a) aMG antiserum showing positive fluorescence in the superficial epithelium and the deep glands; (b) the same antiserum as in (a) absorbed by 250 mg/ml of crude extracts of mucinous ovarian cyst fluid. Note the absence of fluorescence in the superficial epithelium; (c) aMO antiserum showing positive fluorescence only in the superficial epithelium.

(c) *Colonic mucosa.*—Non-absorbed aMO antiserum did not stain the colonic mucosa.

Non-absorbed aMG antiserum stained the Lieberkühn glands of the colonic mucosa. After absorption with the colonic extract (250 mg/ml) this aMG antiserum did not stain the colonic mucosa, but was still positive on the gastric mucosa.

### DISCUSSION

We have proved in this paper the existence of gastric antigens which have the same reaction as antigens present in the ovarian mucinous cyst fluids. The high molecular weight of these components ($>10^6$ daltons), their viscosity in isotonic solutions and their solubility in 2M NaCl, make it likely that they are mucoproteins. Other evidence favouring this hypothesis is the presence of these antigens in the mucinous fluids of some ovarian cysts, and their localization by immunofluorescence in the mucus-producing cells of gastric mucosa.

One of these antigens was also found in colonic mucosa. Hence it could be identical to the antigen observed by immunofluorescence as common to ovarian cysts and colonic mucosa (Nairn *et al.*, 1971). Furthermore, we found that 2/6 mucinous ovarian cysts contained another antigen, able to react with antiserum against a colonic mucosa sulphoglycopeptide (Bara *et al.*, to be published). The relationship of this latter antigen to that described by Nairn is a matter for discussion.

The data obtained by McNeil *et al.* (1969) are worth discussion here. These authors prepared an antiserum against

FIG. 8.—Frozen section from mucinous ovarian cyst. aMG antiserum showing positive fluorescence in epithelial tissue and in the lumen of the cyst (× 250).

ovarian mucinous cyst fluid that gave them 2 or 3 precipitin lines with a pool of colonic tumours in immunodiffusion. As some colonic tumours contain, in our experiments (Bara *et al.*, to be published) HMW antigens of gastric type, as shown also by Kawasaki and Kimoto (1974), it is not unlikely that McNeil's antigens are identical to some of ours.

The antigens already isolated from digestible mucosae may be compared to ours. The component described by André and Descos (1975) as a high-molecular-weight antigen of gastric mucosa is probably identical to one of ours, as we used the same method of fractionation. However, André and Descos did not study the localization of their antigen by immunofluorescence, nor did they check its presence in ovarian cysts. The same holds true for the high-molecular-weight colonic glycoprotein (CMA) isolated by Gold and Miller (1974) that could be compared to the antigen we described as common to colonic and gastric mucosae. Its tissue localization and its presence in ovarian cysts were not investigated.

Finally, we have to stress that only mucinous ovarian cysts contain these antigens in common with gastric mucosa. Until now it was admitted that the epithelium that coats ovarian mucinous cysts has a morphology analogous to that of intestinal mucosa. This morphological similarity has its immunological counterpart, since there are antigen(s) common to these cysts and to colonic mucosa (Nairn *et al.*, 1971). The new fact we report here is the demonstration of several antigens common to ovarian mucinous cysts and gastric mucosa, besides that (or those) already known to be common to mucinous ovarian cysts and colonic mucosa.

The authors are grateful to Dr Hirsch-Marie for the research of antibodies against pepsinogens, Dr J. P. Wolff for the gift of several ovarian cysts and Mrs Vicomte for preparing frozen sections of tissues.

### REFERENCES

ANDRÉ, F. & DESCOS, F. (1975) Purification d'une Glycoproteine Gastrique Humaine et Étude de ses Composants Glucidiques. *Biochem. biophys. Acta*, **386**, 129.

AVRAMEAS, S. & TERNYNCK, T. (1969) The Cross Linking of Proteins with Glutaraldehyde and its Use for the Preparation of Immunoadsorbents. *Immunochemistry*, **6**, 53.

BUFFE, D. & RIMBAUT, C. (1975) α2H Globulin, a Hepatic Glycoferroprotein: Characterization and Clinical Significance. *Ann. N.Y. Acad. Sci.*, **259**, 417.

GOLD, D. V. & MILLER, F. (1974) Characterization of Human Colonic Mucoprotein Antigen. *Immunochemistry*, **11**, 369.

GOLD, P. & FREEDMAN, S. O. (1965) Demonstration of Tumor-specific Antigens in Human Colonic Carcinomata by Immunological Tolerance and Absorption Techniques. *J. exp. Med.*, **121**, 439.

KAWASAKI, H. & KIMOTO, E. (1974) Mucosal Glycoproteins in Carcinoma Cells of Gastrointestinal Tract, as Detected by Immunofluorescence Technique. *Acta path. jap.*, **24**, 481.

LOISILLIER, F., POZZUOLI, R. & BURTIN, P. (1971) Mesures Comparatives de la Teneur en Lactotransferrine dans Certains Organes. *Pathol. Biol.*, **19**, 167.

McNEIL, C., LADLE, J. N., HELMICK, W. M., TRENTELMAN, E. & WENTZ, M. W. (1969) An Antiserum to Ovarian Mucinous Cyst Fluid with Colon Cancer Specificity. *Cancer Res.*, **29**, 1535.

NAIRN, R. C., WALLACE, A. C. & GUU, E. P. G. (1971) Intestinal Antigenicity of Ovarian Mucinous Cystadenomas. *Br. J. Cancer*, **25**, 276.

VON KLEIST, S., KING, M. & BURTIN, P. (1974) Characterization of a Normal Tissular Antigen Extracted from Human Colonic Tumors. *Immunochemistry*, **11**, 249.

VON KLEIST, S., CHAVANEL, G. & BURTIN, P. (1972) Identification of an Antigen from Normal Human Tissue that Crossreacts with the Carcinoembryonic Antigen. *Proc. natn. Acad. Sci. U.S.A.*, **69**, 2492.